# Production and Characterization of RNA Aptamers Specific for Amyloid Fibril Epitopes

**DOI:** 10.1074/jbc.M703679200

**Published:** 2020-09-21

**Authors:** David H.J. Bunka, Benjamin J. Mantle, Isobel J. Morten, Glenys A. Tennent, Sheena E. Radford, Peter G. Stockley

**Affiliations:** ‡ Astbury Centre for Structural Molecular Biology, University of Leeds, Leeds LS2 9JT; § Centre for Amyloidosis and Acute Phase Proteins, Hampstead Campus, University College London, London NW3 2PF, United Kingdom

## Abstract

One of the most fascinating features of amyloid fibrils is their generic cross-β architecture that can be formed from many different and completely unrelated proteins. Nonetheless, amyloid fibrils with diverse structural and phenotypic properties can form, both *in vivo* and *in vitro*, from the same protein sequence. Here, we have exploited the power of RNA selection techniques to isolate small, structured, single-stranded RNA molecules known as aptamers that were targeted specifically to amyloid-like fibrils formed *in vitro* from β_2_-microglobulin (β_2_m), the amyloid fibril protein associated with dialysis-related amyloidosis. The aptamers bind with high affinity (apparent *K_D_* ∼ nm) to β_2_m fibrils with diverse morphologies generated under different conditions *in vitro*, as well as to amyloid fibrils isolated from tissues of dialysis-related amyloidosis patients, demonstrating that they can detect conserved epitopes between different fibrillar species of β_2_m. Interestingly, the aptamers also recognize some other, but not all, amyloid fibrils generated *in vitro* or isolated from *ex vivo* sources. Based on these observations, we have shown that although amyloid fibrils share many common structural properties, they also have features that are unique to individual fibril types.

A number of proteins and peptides undergo specific aggregation *in vivo*, leading to a range of pathological disorders, collectively known as amyloidoses (1, 2). These diseases are characterized by the deposition of normally soluble proteins or peptides into insoluble fibrillar arrays with a cross-β architecture (1, 2). About 30 different proteins have been identified as the fibrillar component of human amyloid deposits to date, although studies have shown that amyloid-like fibrils can be generated *in vitro* from virtually any protein sequence under suitable experimental conditions (3, 4). Remarkably, although amyloid and amyloid-like fibrils are formed from diverse precursor proteins with unrelated primary sequences and tertiary folds, they all adopt a cross-β molecular architecture, identified by x-ray fiber diffraction (5, 6) and bind a number of histological dyes, such as thioflavin T (ThT) and Congo Red (7, 8), the latter showing a characteristic optical anisotropy with apple-green birefringence when viewed using cross-polarized light (9). The ubiquitous ability of amyloid fibrils to bind serum amyloid P component (10, 11), glycosaminoglycans and apolipoprotein E (12), together with the finding that antibodies (*e.g.* WO1) raised against Aβ-(1-40) amyloid fibrils are also able to recognize amyloid fibrils formed from a wide variety of proteins and peptides, unrelated in sequence and structure (13), reinforces the view that amyloid fibrils possess a common core structure. Despite the immense interest in this field (1, 14), the mechanism of how normally soluble proteins or peptides are transformed into the ordered cross-β structure of amyloid is largely unknown, although partial denaturation of the native protein, or folding of disordered states to a partially folded conformer, are thought to be a critical first step (15, 16).

The aggregation and deposition *in vivo* of β_2_-microglobulin (β_2_m)^3^ into amyloid fibrils occurs as a complication of long-term hemodialysis for end-stage renal failure leading to dialysis-related amyloidosis (DRA) (17), a disorder that currently affects more than 750,000 people worldwide (18, 19). β_2_m normally forms the noncovalently bound light chain of the class I major histocompatability complex. As part of its normal catabolic cycle, β_2_m dissociates from the heavy chain of the class I major histocompatability complex that is displayed on the surface of all nucleated cells, whereupon it is carried in the plasma to the kidneys where it is filtered in the glomeruli and degraded in the proximal tubules (20). As a consequence of renal failure, the concentration of circulating β_2_m increases by up to 60-fold (21). Through a mechanism that is not fully understood, the full-length, wild-type, disulfide oxidized protein then self-assembles to form amyloid fibrils that typically deposit in and around osteoarticular sites (22, 23). Despite the identification of β_2_m as the causative agent of DRA more than 20 years ago (17), there are currently no therapies for the disorder other than organ transplantation or other surgical procedures (24), an option that is only available for a minority of patients. New effective therapies for DRA, and amyloidosis, in general, are urgently required.

Developing effective therapies against amyloid disease remains an immense challenge (1, 25). Ligands able to bind and stabilize the native state of an amyloidogenic protein provide one such potential strategy, at least for amyloidogenic proteins displaying enzyme active sites or ligand binding sites (26, 27, 28). Alternative strategies include inhibition of amyloid fibrillogenesis by novel glycosaminoglycan inhibitors, fibril disassembly by β-sheet breaker peptides or enhancement of clearance of existing amyloid deposits, either by immunotherapy or small molecule inhibitors of serum amyloid P component (reviewed in Ref. 29). Alternative strategies based on antibody therapy also have potential (30), although stimulation of the autoimmune response provides future challenges to further developments in this area (31).

A novel, promising class of compounds with potentials as research tools and for the diagnosis or therapy of amyloidosis are RNA or DNA aptamers, small structured polynucleotide sequences that can be isolated by *in vitro* selection from randomized oligonucleotide libraries (32, 33, 34, 35). Indeed, RNA aptamers have already been generated against amyloid-like fibrils formed from Aβ-(1-40) (36), and the disease-associated conformation of the prion protein (PrP) (37), the latter inhibiting conversion *in vitro* of monomeric PrP to the infectious scrapie form (PrP^Sc^) (38, 39, 40). Aptamers have distinct advantages over antibodies as potential therapeutics and diagnostics as they are significantly smaller, can be isolated rapidly *in vitro* and modified to include functional groups including chromophores, fluorophores, radiolabels, or novel functional groups. In addition, aptamers do not carry the secondary functional signals of antibodies, such as complement fixation, and do not elicit a dramatic immune response (35, 41). The first aptamer-based drugs are beginning to appear in the clinic (42) (reviewed in Ref. 43). Aptamers thus provide exciting and currently untapped opportunities for exploitation in amyloid disease.

Exploiting our ability to form amyloid-like fibrils of β_2_m *in vitro* of different morphological types (44), we describe the selection of high-affinity RNA aptamers capable of recognizing such fibrils, and show that these aptamers also bind to β_2_m amyloid fibrils formed *in vivo* in patients suffering from DRA. In parallel, we demonstrate cross-reactivity of these aptamers with amyloid fibrils generated from other proteins, either produced *in vitro* or isolated *ex vivo*, revealing evidence for both shared and unique epitopes among different fibril types.

## EXPERIMENTAL PROCEDURES

*Expression and Purification of Recombinant* β*_2_m*—Expression and purification of full-length recombinant wild-type β_2_m was carried out as previously described (45).

*Preparation of* β*_2_m Fibrils in Vitro*—Amyloid-like fibrils were generated *in vitro* using an adaptation of established protocols (44, 46). Lyophilized β_2_m was resuspended in Milli-Q water (pH 7.0, Millipore Ltd) to ∼5mgml^−1^ and filtered (0.2-μm cellulose acetate filter; Sartorius) before use. Fibrils with different morphologies were then formed by incubation of the protein under different buffer conditions. Short (∼400 nm) curved “worm-like” (WL) fibrils were formed by diluting β_2_m to1mgml^−1^ in a final concentration of 25 mm sodium acetate, 25 mm sodium phosphate buffer (Buffer A), pH 3.6, containing 250 mm NaCl and incubating the solution at 37 °C for 7 days with shaking at 200 rpm. Small “rod-like” fibrils were formed in Buffer A, pH 3.6 (37 °C, 7 days), without NaCl or agitation. Fibrils with a long straight (LS) morphology, typical of amyloid fibrils isolated *ex vivo* from tissues of patients suffering from DRA (47), were formed by incubating β_2_m in Buffer A, without NaCl, at pH 2.5 for 7 days at 37 °C, with shaking at 200 rpm (these fibrils are denoted “LS 2.5”). Finally, fibrils were formed at neutral pH (37 °C, 7 days) using a seeded reaction in which fibrils formed at pH 2.5 were stabilized by the addition of heparin (46) and were then frozen (-20 °C) to create fragmented fibril seeds. These seeds were then elongated at pH 7.0 by adding 10% (w/w) of seed to a solution of monomeric β_2_m (1 mg ml^−1^) in Buffer A at pH 7.0 (lacking NaCl) at 37 °C with agitation (46). Fibrillogenesis was monitored by ThT fluorescence and negative stain TEM as previously described (48).

*Biotinylation and Immobilization of* β*_2_m*—Isolated pure monomeric β_2_m (∼1 mg) and β_2_m amyloid fibrils produced *in vitro* (∼1 mg of each morphological type) were biotinylated (EZLink™ Sulfo-NHS-LC-LC-biotin, Pierce), at neutral pH using ∼20-fold molar excess of biotin over the total protein concentration, according to the manufacturer's protocol. The reaction time was reduced to 10 min for fibril samples to avoid disassembly of the fibrils formed under acidic conditions (which is known to occur at neutral pH (46, 49)). ThT assays (48) confirmed that fibrils were present at every step of the biotinylation reaction. Electrospray ionization mass spectrometry (ESI MS; LCT Premier, Micromass UK Ltd.) of monomer and fibrils (first depolymerized by incubation at neutral pH (46)) confirmed that biotinylated β_2_m was present in both fibrillar and monomeric forms with, on average, 1 or 2 biotinyl groups incorporated per monomer. Mass spectroscopy/mass spectroscopy sequencing (Tandem Q-Tof, Micromass UK Ltd.) demonstrated that the modifications for both monomer and fibril typically occurred at the N terminus and in residues in the native A and G strands (Lys^7^ and Lys^92^ for the monomer; Arg^13^, Lys^92^, and Lys^95^ for WL fibrils). The biotinylated samples were then immobilized on 1-μm streptavidin-coated microspheres (Dynabeads™, Dynal Biotech), as described previously (50).

*In Vitro Selection*—A Biomek 2000 laboratory automation work station (Beckman Coulter) was used to perform simultaneous iterative *in vitro* selections with a synthetic combinatorial N60 RNA library (∼10^15^ sequences) directed against the three β_2_m targets: monomeric β_2_m at low pH (designated LM), WL fibrils, and LS fibrils, using minor modifications of the protocols described previously (50). Selections were carried out in buffers appropriate to the target: Buffer A, pH 3.6 for the low pH monomer and WL fibrils, and Buffer A, pH 2.5 for LS fibrils. Negative selections were carried out on the 1st, 5th, and 10th selection rounds. Aptamers directed against monomeric β_2_m and LS fibrils were then counterselected against underivatized Dynabeads, whereas aptamers directed against WL fibrils were counterselected against beads carrying biotinylated monomeric β_2_m at pH 3.6. Samples from the reverse transcriptase-PCR products were taken at the end of each round and analyzed by native PAGE to confirm the isolation of products for the next round of selection.

*Binding Specificities of Anti-*β*_2_m Aptamers Measured Using Surface Plasmon Resonance*—The binding specificities of the three selected anti-β_2_m aptamers were studied by surface plasmon resonance (SPR) using a BIAcore 3000 instrument with the biotinylated selection targets immobilized on the sensor chip (SA using Buffer A, pH 3.6, containing 250 mm NaCl as running buffer and diluent). Aptamer RNA was dialyzed into the same running buffer before injection across the surface in the same buffer to minimize bulk refractive index effects. Flow rates and the regeneration of chip surfaces were as described previously (51). Data were analyzed using the manufacturer's software (BIAevaluation).

*Dot Blot Binding Assays*—β_2_m fibrils were pelleted by centrifugation (13,000 × *g* for 5 min), washed with buffer A at the appropriate pH and ionic strength to ensure all monomer was removed, resuspended in the same buffer to ∼1mgml^−1^ (based on initial monomer concentration and assuming 100% fibril yield), and diluted to a range of concentrations (1-1000 μg ml^−1^). A 2-μl aliquot of each sample was then taken and spotted onto nitrocellulose membranes (0.45 μm, Hybond ECL, Amersham Biosciences). Once dry, the membranes were stored (4 °C) in sealed plastic bags until required. For the binding assay (conducted at 21 °C), each membrane was pre-wetted in 10 ml of Buffer A, pH 3.6 or 7.0, containing 250 mm or 1 m NaCl, by rolling in a Falcon tube for 5 min. To block nonspecific binding, ultrapure bovine serum albumin (Ambion Inc.) and yeast tRNA (Ambion Inc.) were each added to a final concentration of 10 μg ml^−1^ and after a 10-min incubation, ∼5 × 10^6^ cpm of ^32^P-labeled RNA ([^32^P]RNA) (52) was added and incubated for an additional 10 min. Membranes were then repeatedly washed in 20 ml of buffer for 10-min cycles until no significant radioactivity was detected in the buffer. The membranes were then air dried and wrapped in cling-film before exposure to autoradiography film (Biomax MR film, Kodak) for 1 h (21 °C). Autoradiographs were scanned (FujiFilm FLA-5100) and spot intensities compared using AIDA software. Membranes were also stained with Deep Purple™ total protein stain (GE Healthcare) according to the manufacturer's instructions.

*Dot Blot Cross-reactivity Assays*—To test whether aptamers raised against β_2_m fibrils produced *in vitro* cross-react with other types of amyloid fibrils, dot blots were prepared, as described above, with amyloid-like fibrils formed from Aβ-(1-40), transthyretin (human, Sigma), lysozyme (hen egg, Sigma), and apomyoglobin (horse heart, Sigma) produced *in vitro* according to established protocols (53, 54, 55, 56). Blots were prepared also with *ex vivo* amyloid fibrils isolated according to a standard protocol (57) and without further purification, from unfixed frozen amyloidotic tissues (obtained with informed consent in accordance with the Declaration of Helsinki) of patients with DRA, and hereditary systemic amyloidoses of lysozyme (D76H variant) and transthyretin (F33L variant) (58). All blots were then incubated with radiolabeled aptamers as described above.

## RESULTS

**FIGURE 1:**
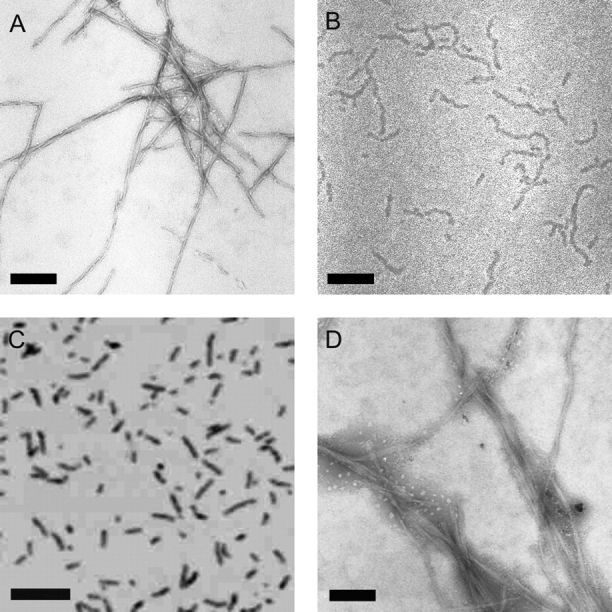
**Images of** β**_2_m fibrils.***A*, long-straight (LS 2.5) fibrils formed from β_2_m at pH 2.5; *B*, WL fibrils formed at pH 3.6; *C*, rod-like fibrils formed at pH 3.6; *D*, LS fibrils formed at pH 7.0. *A, B*, and *D*, negatively stained TEM images; *C*, tapping mode atomic force microscope image. Each *scale bar* represents 200 nm. Note, LS 2.5 and LS 7.0 fibrils are morphologically identical but the latter tend to stick together in negative stain.

*Selection of Anti-*β*_2_m Amyloid Fibril Aptamers*—A panel of amyloid-like fibrils of β_2_m was formed by incubating full-length, wild-type human β_2_m under different conditions *in vitro*, to produce fibrils with distinct morphologies (Fig. 1). At low pH, fibrils form rapidly and spontaneously without the need to seed polymerization (44, 59). At low pH and low ionic strength (pH 2.5; I < 100 mm), β_2_m rapidly self-assembles from an initially highly unfolded state (60) to form long-straight fibrils (LS 2.5) (Fig. 1A), which display the defining characteristics of amyloid, namely green birefringence in cross-polarized light microscopy after binding to Congo Red (61) and significant specific calcium-dependent binding by serum amyloid P component (46). These fibrils bind also to monoclonal anti-amyloid antibodies (WO1) (44), induce the characteristic spectral red shift after incubation with ThT, and give rise to an x-ray fiber diffraction pattern consistent with a cross-β fibril structure (44, 45, 61). Incubation of β_2_m at higher pH and ionic strength (pH 3.6; >250 mm added NaCl), results in the formation of curved or nodular fibrils 200-400 nm in length, that have a worm-like appearance (Fig. 1B) and also display many of the hallmarks of amyloid (61), although they are distinct because they cannot progress directly to mature fibrils (44). Under these conditions, assembly is initiated from a partially folded conformer that retains at least 5 of the 7 β-strands that comprise the native structure (62). Rod-like fibrils are formed under the same conditions as WL fibrils, but in the absence of added NaCl and without agitation (44). These short (∼20-50 nm) particles have been shown to be precursors of WL fibrils, but are not able to assemble further into LS fibrils (44) (Fig. 1C). Finally, fibrils with an LS morphology were generated at physiological pH and ionic strength conditions (named LS 7.0) by seeding growth with fibrils originally generated at pH 2.5. These were stabilized at pH 7.0 by the addition of heparin, fragmented to form seeds by freeze-thaw cycles, and subsequently elongated with monomeric β_2_m at pH 7.0 (46) (Fig. 1D). Under these conditions, β_2_m fibrillogenesis proceeds from a partially folded conformer containing a non-native proline isomer at residue 32 (16), and requires the participation of biological factors pertinent to the joint environment in which these fibrils deposit *in vivo* (46, 63).

Aptamer selection targets were prepared by biotinylation of β_2_m monomer, WL fibrils, or LS fibrils formed at pH 2.5. Each species was immobilized onto streptavidin-coated microspheres and used as the targets in 10 rounds of *in vitro* selection with an N60 degenerate RNA library (see “Experimental Procedures”). Selections were carried out using buffer conditions appropriate for each target to ensure that fibril samples remained assembled during each stage. Aptamers in the WL fibril selections were counterselected against low pH monomer and unmodified microspheres on three of the selection rounds. The aptamers raised against LS fibrils or low pH monomers were counterselected against microspheres only.

*Identification of Specific Epitopes on Different* β*_2_m Fibril Types*—After 10 cycles of *in vitro* selection the three aptamer pools were cloned and ∼20 of each sequenced. As expected after 10 rounds of selection, the pools contain a number of distinct sequences that cluster into families based on short (<10 base) sequence motif matches, with some individuals showing matches in several families (supplemental Fig. S1). Although partial consensus sequences are clearly present, confirming that selection had occurred, there was no one obviously dominant epitope binding consensus. Comparison of these sequences with those reported elsewhere for either anti-Aβ-(1-40) (36) or anti-PrP aptamers (39) did not show significant sequence motif matches, suggesting that the aptamers raised are specific to the β_2_m selection targets.

**FIGURE 2:**
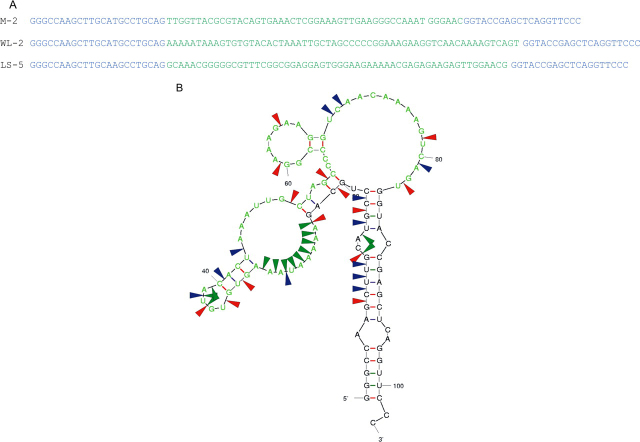
**RNA sequences of representative individual aptamers from each selection.***A*, the selected region is shown in *green* and the fixed primer regions in *blue. M-2*, aptamer selected against monomeric β_2_m at pH 3.6; *WL-2*, aptamer selected against WL fibrils at pH 3.6; *LS-5*, aptamer selected against LS fibrils at pH 2.5. The Mfold-predicted, secondary structure of aptamer WL-2 was confirmed by enzymatic solution structure probing (*B*). The random region is again shown in *green*; sites of cleavage by RNase T1 (G specific), RNase A (U and C specific), and S1 (single strand specific) nucleases are shown by *red, blue*, and *green arrows*, respectively.

To confirm that these aptamers had the desired target binding properties preliminary binding data were obtained using SPR (not shown). One individual from each selected pool (M-2 for the anti-low pH monomer; WL-2 for the anti-WL fibrils and LS-5 for the anti-LS 2.5 selections, respectively; see Fig. 2B and supplemental materials) was then chosen at random for further analysis. Mfold software (64, 65) was also then used to derive putative secondary structures for these aptamers (see Fig. 2B and supplemental Figs. S2 and S3) and in one case the structure was confirmed directly by enzymatic structure probing (Fig. 2B). These structures show that the selected regions contain extended interrupted stem-loop structures some of which are dependent on base pairing with the fixed templates regions, which is a common finding for such species.

**FIGURE 3:**
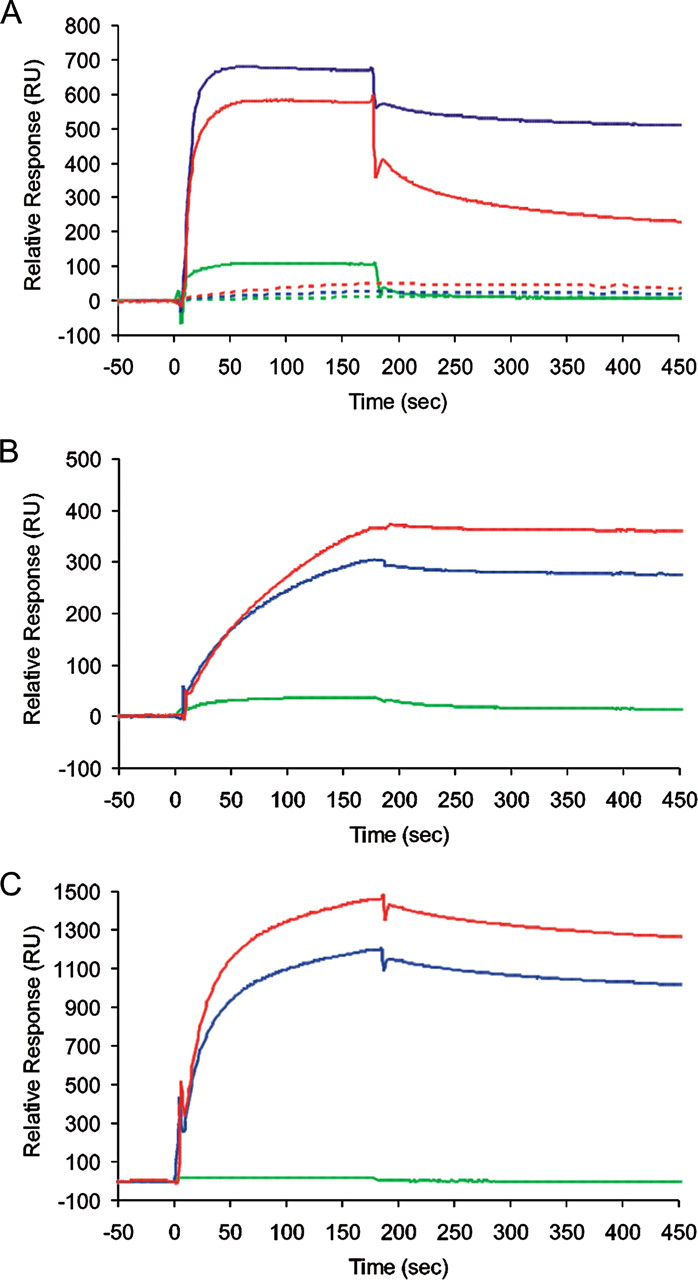
**Surface plasmon resonance experiments demonstrating specificity and cross-reactivity of representative aptamers with different targets.** Sensorgrams were generated by passing individual aptamer RNA (0.5 μm) from the anti-monomer (M-2) (*A*), anti-LS, pH 2.5 (LS-5) (*B*), or anti-WL (WL-2) (*C*) selections, across flow cells derivatized with monomeric β_2_m (*green*), WL fibrils (*blue*), or LS fibrils (pH 2.5) (*red*). *Panel A* also shows the results of injecting equivalent amounts of the unselected naïve starting pool of RNA across these targets (*dotted lines*). All sensorgrams were recorded at a flow rate of 10 μl min^−1^ in Buffer A, pH 3.6, containing 250 mm NaCl at 25 °C. All immobilized targets were stable over the time course of the experiment. Biotinylated monomeric β_2_m, WL, or LS fibrils were derivatized onto separate flow cells by injecting 50 μl of 50 μg ml^−1^ β_2_m (or the equivalent monomer concentration in the fibrillar samples), using a flow rate of 10 μl min^−1^. During the protein injection, sensorgrams reached a “plateau” beyond which it was impossible to achieve any further levels of derivatization, even at higher concentrations. This is consistent with steric hindrance in accessing the streptavidin on the sensorchip by the macroscopic fibrillar targets. In total ∼200 response units (RU) monomeric β_2_m, ∼2800 RU of WL fibrils, and ∼1300 RU of LS fibrils were immobilized. All sensorgrams were corrected for nonspecific binding and refractive index changes by subtracting the signals of an equivalent aptamer injection across an adjacent, underivatized flow cell.

To characterize the binding of these aptamers to each target, and to determine their ability to discriminate between different targets, binding to the different fibril types or low pH monomer was assayed by SPR (Fig. 3). This figure also shows the results of passing the unselected naïve starting pool across each target. There are large signal differences between this pool and the selected individual aptamers confirming that the responses seen are due to specific binding and not simply that fibrils are “sticky” for RNA. Sensorgrams generated by passing the anti-low pH monomer aptamer, M-2, across flow cells immobilized with monomeric β_2_m, or the WL or LS fibrils, clearly show binding of this aptamer to all three forms of β_2_m, demonstrating the conservation of at least one epitope between these species (Fig. 3A). Interestingly, although binding of these aptamers to both the LS and WL fibrils resulted in significantly higher responses than to the monomer, the increased SPR signal was not simply proportional to the amount of β_2_m immobilized in each case, suggesting that the epitope recognized is partially occluded within the fibrils relative to the low pH monomer. Aptamer dissociation from the fibrils was also very slow (see supplemental Fig. S4), implying that rebinding was significant, as would be expected with such macroscopic targets, presumably because the repetitive structure of amyloid results in an array of binding sites in close proximity.

Unfortunately, despite extensive efforts, it proved impossible to find conditions that would allow us to regenerate the derivatized sensor chips without losing some of the immobilized fibrillar targets. To avoid problems with interpretation we chose to run a number of separate experiments each with single analyte injections. These were reproducible in form and gave consistent kinetic constants (see supplemental Table S1). Sensorgrams for binding of M2 to the cognate low pH monomer fitted to a simple 1:1 binding model, yielded an apparent *K_D_* ≈ 10 nm. Sensorgrams for the aptamers binding to the fibrillar targets could not be fitted to this model due to the increasing extent of re-binding in the dissociation phase. To derive apparent affinities in these cases, we initially determined the apparent dissociation rates over a 30-s period, conditions where pseudo-first order behavior occurs (66), and then used these values in the instrument software to derive the apparent association rates and hence the apparent equilibrium constants, assuming a 1:1 binding model. The resultant χ^2^ values (supplemental Table S1) suggest that the data were good fits to this model, which yielded apparent *K_D_* values of ∼4 and ∼7nm for binding of M2 to the WL and LS fibril targets, respectively, with χ^2^ values ∼1. These data demonstrate, therefore, that the selections had generated an aptamer capable of tight binding to all three forms of β_2_m, suggesting that the WL and LS fibrils share at least one epitope in common with the low pH monomer.

In contrast with the data obtained using the aptamer M2, binding of the anti-LS aptamer, LS-5, to the three protein targets demonstrated that this aptamer was more specific for the fibrillar forms of β_2_m compared with their monomeric counterpart (Fig. 3B). Fitting the resulting sensorgrams revealed that LS-5 binds to both the LS and WL fibrils with ∼20 nm apparent *K_D_* values, whereas the equivalent affinity for monomeric β_2_m was only ∼200 nm. Interestingly, the anti-WL fibril aptamer, WL-2, also binds to both LS and WL fibrils with apparent *K_D_* values of ∼10 nm, whereas binding to monomeric β_2_m was much weaker (apparent *K_D_* ≈∼5 μm). Because the latter aptamer was counterselected against the low pH monomer, the results demonstrate the success of this step in enhancing the specificity for the fibrillar targets. WL-2 binds also to WL and LS fibrils with an on-rate that is much faster than the binding of LS-5 to the same fibrillar targets (*cf.* Fig. 3, B and C), suggesting that although these aptamers recognize an epitope present in both WL and LS fibrils, the epitope for each aptamer must be either distinct or differentially accessible in the different fibril forms. Because there is only weak binding of WL-2 to the low pH monomer this epitope is fibril-specific. Finally, because the LS fibril target binds the most aptamer irrespective of the type of fibril used as selection target, and despite the fact that there is less than half the mass of this target on the sensor chip compared with the WL fibril, the data demonstrate that the LS fibrils display the highest avidity for the RNA sequences selected.

**FIGURE 4:**
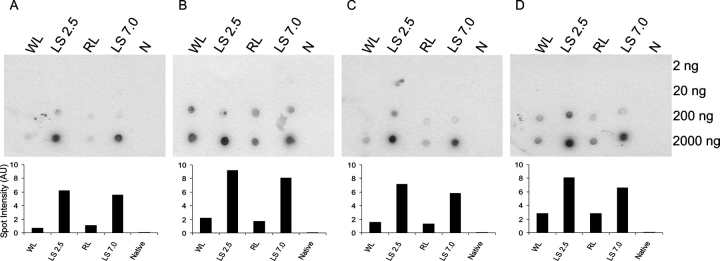
**Dot blots displaying aptamer specificity against different forms of** β**_2_m.** Increasing amounts (2-2000 ng in serial 10-fold dilutions) of β_2_m samples (annotated *above* each blot) were spotted onto nitrocellulose membranes. The blots were then incubated at pH 7.0 with [^32^P]RNA aptamer pools selected against low pH monomer (*A*); LS pH 2.5 fibrils (*B*); WL fibrils (*C*); or the individual aptamer WL-2 (*D*) as described under “Experimental Procedures.” Densitometry of the spot intensity at the highest protein concentration is shown *beneath* each blot. The incubation buffer contained 1 m NaCl to remove nonspecific binding.

*Developing a Rapid Comparative Binding Screen*—To screen the binding specificities of the aptamers in more detail, and specifically to test the cross-reactivity of the aptamers selected against β_2_m fibrils with amyloid fibrils generated from other proteins, a high throughput method based on a dot blot format was developed. The dot blot assay provides only semiquantitative comparisons, but is rapid, easy to perform and, most importantly, *ex vivo* amyloid fibril isolates can be analyzed without additional purification. To develop such an assay, a dilution series ranging from 2 to 2000 ng of different conformational states of β_2_m (native monomer, pH 7.0 (N), LS fibrils formed at pH 7.0 (LS pH 7) or 2.5 (LS pH 2.5), and rod-like or WL fibrils formed at pH 3.6) was spotted onto a nitrocellulose membrane, dried, and incubated with [^32^P]RNAs from one of the aptamer 10th round selection pools or the individual aptamer WL-2. Spots were then visualized by autoradiography and binding quantified by densitometry (Fig. 4).

Initial experiments in which binding to the blots was performed under identical conditions to those used in the SPR experiments (pH 3.6, 250 mm NaCl) resulted in significant non-specific binding, especially for the pools. The problem was alleviated by the inclusion of 1 m NaCl in the binding buffer, presumably because nonspecific binding of the negatively charged RNAs to the positively charged β_2_m is reduced at the higher ionic strength. This higher ionic strength may be required to ensure specific binding in the dot blot assays because of inefficiencies in sample washing of the membrane compared with the SPR assay. In addition to this, increasing the pH of the dot blot incubation to 7.0, where β_2_m carries a net negative charge, removed all traces of nonspecific binding as confirmed by the observation that no binding to any target was observed using a poly(U) RNA or the unselected RNA starting pool (data not shown). In further support of this view, none of the aptamers bind to the native (N) monomeric β_2_m at pH 7.0 (Fig. 4). Importantly, previous experiments have shown that the fibrils are stable on the membrane surface for the time course of these experiments (44, 46). These results demonstrate, therefore, that the dot blot assay can be used to screen the binding of RNA aptamers to different protein targets and that the interactions are not primarily electrostatic in nature.

As expected based on the results of the SPR experiments, the dot blot assays indicated that the aptamers raised against either the LS or WL fibrils are able to bind to all fibrillar forms of β_2_m (Fig. 4, B-D), confirming the presence of common epitopes in fibrils with different morphology. In all cases the LS fibrils bind the aptamers with highest affinity (dot intensity), even if they were originally raised against the WL fibril target. Also as expected based on the fact that the LS fibrils at pH 7.0 were formed by seeding growth with fragmented fibrils formed initially at pH 2.5, binding of the aptamers to these two fibril types, in all cases, was very similar. Given that strong binding to fibrils with a LS morphology is a common property of all the aptamers tested, the results suggest that this fibril type possesses one or more epitope(s) in common with the WL fibrils, as well as with the low pH monomer. All of these sites(s) have a high avidity for RNA. Interestingly, aptamers raised against partially folded monomeric β_2_m at low pH bind to both LS and WL fibrils, consistent with these forms of β_2_m sharing common structural properties (Fig. 4A). None of the aptamers are able to bind to native monomer, confirming the specificity of the interactions observed. Importantly, this observation also suggests that the native folded species does not share epitopes in common with the fibrillar states or their precursor. Conversely, it implies that there are unique native epitopes that could be targeted for future aptamer selections.

**FIGURE 5:**
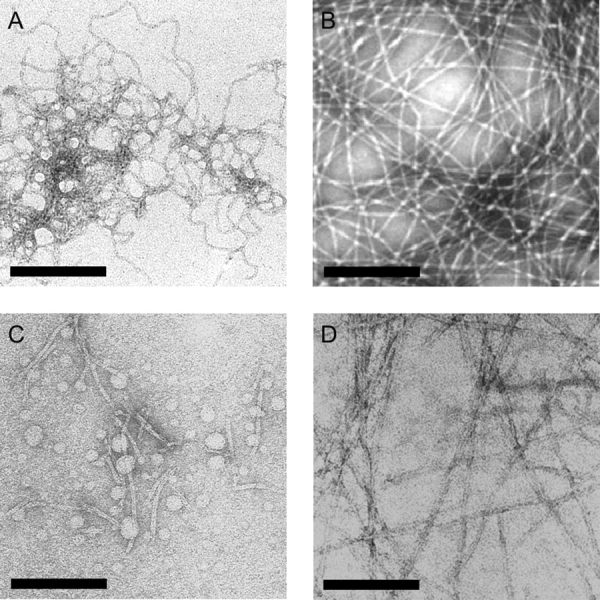
**Negative stain TEM images of amyloid-like fibrils formed*****in vitro**. A*, apomyoglobin; *B*, Aβ-(1-40); *C*, transthyretin; or *D*, lysozyme. *Scale bar* represents 200 nm.

**FIGURE 6:**
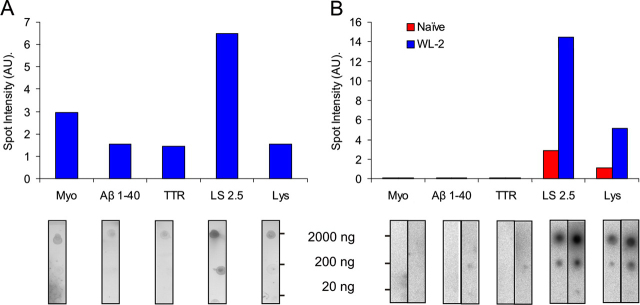
**Cross-reactivity of aptamers with amyloid-like fibrils formed from different proteins*****in vitro***. A dilution series of 20-2000 ng of amyloid-like fibrils made from apomyoglobin (*Myo*), Aβ-(1-40), transthyretin (*TTR*), β_2_m (*LS 2.5*), or lysozyme (*Lys*) was spotted onto replicate nitrocellulose membranes and visualized with Deep Purple total protein stain (*A*), or incubated with [^32^P]RNA (*B*) from either the naïve pool (*red*) or the individual aptamer WL-2 (*blue*). Histograms show spot intensities at the highest protein concentration, quantified by densitometry and corrected for local background.

*Cross-reactivity of Aptamers with Other Amyloid-like Fibrils*—To determine whether the β_2_m fibril-specific epitope(s) identified here are also present in other amyloid-like fibrils, a series of blots was created using amyloid-like fibrils formed *in vitro* from apomyoglobin, Aβ-(1-40), lysozyme, and transthyretin, proteins/peptides unrelated to β_2_m but known to form amyloid-like fibrils *in vitro* (53, 54, 55, 56). The fibrils were characterized by the negative stain TEM (Fig. 5) and ThT fluorescence (data not shown). Importantly for this set of proteins, fibrils with a LS-like morphology (lysozyme, transthyretin, and Aβ-(1-40)), as well as more curvilinear forms, analogous in morphology to the WL fibrils of β_2_m (apomyoglobin), were generated. Fibrils were then purified from each sample by centrifugation and dotted onto nitrocellulose membranes to create a protein dilution series from 20 to 2000 ng. Replicate blots were prepared and incubated with the [^32^P]RNA aptamer WL-2 or the naïve library as a control. To compare aptamer binding to different fibrils, the yield of fibrils in the different growth assays was determined in parallel by staining one blot with Deep Purple total protein stain. Protein concentrations and aptamer binding were then quantified by densitometry (Fig. 6).

The results were striking, revealing that whereas the aptamer WL-2 binds to all fibrillar forms of β_2_m, this aptamer does not recognize amyloid fibrils formed *in vitro* from apomyoglobin, Aβ-(1-40), or transthyretin. Remarkably, however, significant binding is observed to fibrils formed from lysozyme. This is not due to nonspecific interactions, as evidenced by the inability of WL-2 to bind to native monomeric lysozyme and the observation that the naïve pool binds relatively weakly to the lysozyme fibrils under these conditions. Indeed there was no binding to any of the monomeric proteins under these conditions (data not shown) confirming that the epitopes being recognized are fibril specific. Moreover, both lysozyme and apomyoglobin are positively charged at pH 7.0, yet binding is not observed to the latter. Given that lysozyme and β_2_m have unrelated protein sequences, the data suggest that a conformational epitope is shared by both fibrils, but is not present or is inaccessible in fibrils composed of apomyoglobin, Aβ-(1-40), or transthyretin.

**FIGURE 7:**
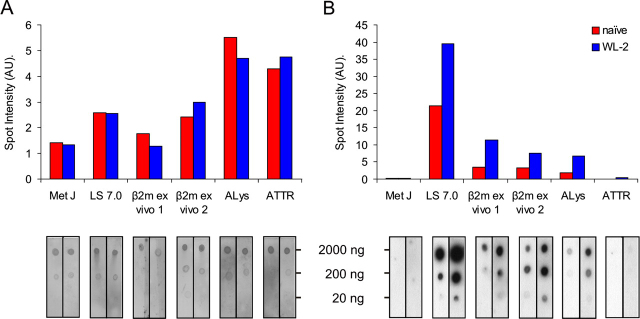
**Aptamers recognize*****ex vivo*****amyloid fibrils.** β_2_m amyloid fibrils isolated from two different patients with DRA (β_2_m *ex vivo*), hereditary systemic lysozyme amyloidosis (*ALys*), or transthyretin amyloidosis (*ATTR*) were dotted onto nitrocellulose membranes and stained for total protein with Deep Purple (*A*) following incubation with [^32^P]RNA (*B*) from the naïve pool (*red*) or aptamer WL-2 (*blue*). Histograms show spot intensities at the highest protein concentration, quantified by densitometry and corrected to local background. The replicates in *A* demonstrate the reproducibility of the protein concentration in different spots and are colored to match the respective dots in *B*.

*Cross-reactivity of Aptamers with ex Vivo Amyloid Fibrils*—Based on the specificity of the aptamers developed against β_2_m fibrils of different morphological type, and their ability to bind selectively to amyloid-like fibrils generated from different proteins *in vitro*, we next questioned whether these aptamers could be used to detect amyloid fibrils associated with human disease. Fibrils were thus isolated from patients with DRA, systemic lysozyme, or transthyretin amyloidosis and analyzed for binding to aptamer WL-2 in dot blot assays. β_2_m fibrils formed *in vitro* from recombinant protein at pH 7.0 and the soluble DNA-binding protein MetJ (67) served as positive and negative controls, respectively. As described above, protein content was assessed by staining with Deep Purple after incubation of the blots with ^32^P-labeled WL-2 aptamer or the naïve pool (Fig. 7). Note, that although *ex vivo* isolates are rich in amyloid fibrils, they also contain traces of other proteins and proteoglycans, so the protein concentrations shown in Fig. 7A are approximate, but serve to validate the comparison of naïve and WL-2 binding. The results show that WL-2 binds to *ex vivo* β_2_m fibrils, demonstrating that the amyloid-like fibrils generated from this protein under both acidic and neutral pH conditions *in vitro* possess structural properties closely related to amyloid associated with DRA. Remarkably, and consistent with the results obtained using synthetic fibrils generated *in vitro*, WL-2 also binds to *ex vivo* lysozyme fibrils, but not *ex vivo* transthyretin fibrils. All of these fibril samples were poorly recognized by aptamers within the naïve pool, again confirming the specificity of their interactions with WL-2. The data for these proteins thus also mirrors the results obtained using synthetic amyloid generated *in vitro*, suggesting at least for these samples that synthetic amyloid and amyloid-like fibrils are structurally closely related.

## DISCUSSION

Devising therapies for amyloid disease provides an immense challenge, not just because of the heterogeneity of the assembly process of different amyloid fibril proteins and the difficulties in identifying key precursor conformations (15, 16), but also because factors responsible for the disease phenotype in different amyloid disorders (and in different patients with the same type of amyloidosis), which may or may not include the different fibril conformational states, are still unknown (1, 14, 68, 69, 70).

Aptamer technology has enormous potential in biology due to the ease with which ligand binding species can be selected *in vitro* from degenerate nucleic acid pools, and the simplicity of handling nucleic acid-based reagents (43, 50, 51). Simple variations in nucleotide chemistry, either during the selection process or within a previously defined aptamer sequence, can be used to endow RNA aptamers with biostability or to label them with electron dense materials, tags for immobilization or fluorophores or radioisotopes for detection (43, 71). RNA and DNA aptamers also have considerable advantages over antibody reagents in that they are smaller and essentially non-immunogenic (35). Here, we have used RNA aptamers to probe the structures of various fibrillar forms of the protein β_2_m generated *in vitro* and *in vivo*. Previous extensive studies have defined the process of amyloid-like fibrillogenesis *in vitro* in this system in great detail (72), making it an ideal model with which to explore whether aptamers can be used to identify common or unique epitopes in the various aggregated forms of the protein.

Our results show clearly that aptamers can be selected that achieve both of these goals. M2, and other aptamers in the 10th round pool, bind to both acid unfolded β_2_m monomer (Fig. 3A), known to be a precursor of WL amyloid fibrils (44, 59), as well as to fibrils with WL and LS morphologies, demonstrating that these species share a common epitope(s). The anti-WL and anti-LS fibril aptamers, WL-2 and LS-5, and others within the 10th round pool selected against each of these fibrils types, bind tightly to both types of fibril, display only very weak binding to the low pH monomer, and do not bind to native β_2_m, demonstrating that these aptamers recognize fibril-specific epitopes. The distinct kinetics and avidity of the different fibril forms for the different aptamers (Fig. 3) implies either that the anti-WL and anti-LS aptamers bind distinct epitopes, or that the solvent exposure of a common epitope differs substantially in the different fibrils types. Alternatively the LS fibrils may simply display a higher frequency of this epitope per unit surface, possibly implying a higher degree of order. Quantification of aptamer binding suggests that these reagents have nanomolar affinities, even though we have not specifically tried to select very tight binders. Remarkably, the fibril-specific aptamer derived from selection against WL fibrils formed *in vitro* from β_2_m at pH 3.6 is able to recognize an apparently conformationally conserved epitope in fibrils of lysozyme, and to discriminate against similar fibrils from other proteins formed *in vitro* and *in vivo* (Figs. 6 and 7). Identification of the conserved and unique epitopes will have to await detailed structural analysis of the amyloid fibrils formed from these very different proteins. Nonetheless, the finding that aptamers are able to bind multiple forms of amyloid fibrils, as well as fibrils formed from some, but not all, unrelated protein sequences, highlights the diversity of fibril architectures built on the common cross-β amyloid fold and offers exciting opportunities for the future in generating generic anti-amyloid aptamers, as well as aptamers able to discriminate between fibril deposits in different amyloid disorders. Because many RNA-binding proteins utilize β-sheets for the recognition of their nucleic acid ligands, an inherent ability of amyloid fibrils to bind RNA aptamers tightly is perhaps not surprising (73, 74). Whereas the full potential of aptamers as therapeutics or diagnostics has yet to be realized, our results demonstrate the power of RNA aptamers for detection and identification of amyloid and, possibly, other protein misfolding diseases. They also pave the way for future experiments to derive aptamers able to bind tightly and specifically to different amyloid precursors (monomers, oligomers, or other protofibrillar forms), or amyloid fibrils with distinct structural properties that give rise to different disease phenotypes (75, 76). For instance, the fact that the native monomeric β_2_m does not share epitopes with either LS or WL fibrils or their precursors suggests that monomer-binding aptamers could be useful therapeutically. The results presented here thus demonstrate the potential utility of RNA aptamers for probing the structures of amyloid fibrils, including the specific recognition and possibly diagnosis of amyloid deposits in different patient samples, using material that is impure, has only low concentrations of fibrils, or contains mixtures of soluble forms of the amyloid protein in concert with other amyloid-associated factors as well as amyloid fibrils themselves.

## Footnotes

3 The abbreviations used are:β_2_m, β_2_-microglobin; DRA, dialysis-related amyloidosis; PrP, prion protein; WL, worm-like; LS, long straight morphology; SPR, surface plasmon resonance; ThT, thioflavin T.
