# Supplementary material for: Production and Characterization of RNA Aptamers Specific for Amyloid Fibril Epitopes
Source: J Biol Chem. 2020 Sep 21;282(47):34500–9. doi: 10.1074/jbc.M703679200 (PMC8782670; doi:10.1074/jbc.M703679200)
Supplement: Supplementary file 1 [file mmc1.pdf]

SUPPLEMENTARY ONLINE MATERIAL

Supplementary Fig. 1. Alignments and Logos for selected aptamer pools. The 'Genebee' program (http://www.genebee.msu.su) was used to identify 10 'supermotifs' within the selected regions of the sequenced aptamers against the various β_2m targets – low pH monomer (A); WL fibrils (B) or LS fibrils (C). The matching sequence regions are shown within each supermotif together with the names of the aptamers. Logos were generated from these alignments using the 'Weblogo' program (http://weblogo.berkeley.edu), and are shown below the respective motifs. Of the 19 sequenced anti-monomeric β_2m aptamers, 1 does not fall into any supermotif. The equivalent figures are 4/18 for the anti-WL fibril aptamers and 2/15 for the anti-LS aptamers.

Supplementary Fig. 2. Secondary structures of the individual aptamers screened. The secondary structure of WL-2 (see Fig. 2) was confirmed by denaturing polyacrylamide gel electrophoresis (A) after enzymatic digestion with RNase A (shown in blue), RNase T1 (shown in red) and S1 nuclease (shown in green). A base hydrolysis ladder (black) and two further marker lanes are also shown. The Mfold predicted secondary structures of aptamers LS-5 & M-2 are shown in B and C, respectively. The random regions are highlighted in green. Arrows on LS-5 (B) indicate bases, specifically protected from RNase A (blue) & RNase T1 (red) upon addition of LS fibrils, see also Supplementary Figure 3.

Supplementary Fig. 3. Nuclease protection assays demonstrate specific binding of LS fibrils. Nuclease protection was used to confirm binding of the cognate target to one of the aptamers, LS-5, using standard methods. Briefly, aptamer LS-5 was digested with RNase A (A) or RNase T1 (B) in the presence of a concentration gradient of LS fibrils (red) or a non-binding control protein, Met J (blue). Example bands showing protection (labelled on the left) were subjected to image densitometry. The resulting plots of relative intensity change (y axis) *vs* gel position (x-axis, arbitrary units) are shown to the right of the autoradiographs and clearly show decreasing band intensities with increasing fibril concentration (red) whilst no consistent changes occur for the MetJ lanes (blue). These demonstrate specific nuclease protection of LS-5 by LS fibrils. A summary of all protected sites is shown alongside the secondary structure plot of Supplementary Figure 2B.

Supplementary Fig. 4. Extended sensorgrams of aptamer WL-2 binding. Aptamer RNA was injected over flow-cells derivatised with WL fibrils (blue) or LS fibrils (red), then left to dissociate over ~35000 seconds. Sensorgrams show that the aptamer RNA does dissociate from fibrils over extended time periods.

Supplementary Table 1. Affinities of the aptamers derived from the SPR assay. Tables show the apparent kinetic parameters for aptamers WL-2, M-2 & LS-5 binding to monomeric β_2m, WL fibrils & LS fibrils. Residual traces (not shown) suggest that 'kinetic fits' fall within 0.5% of experimental data. The derived kinetic parameters for repeated experiments are within 10% of each other, suggesting that these values are highly reproducible.

A

LOCAL SUPERMOTIF 1

```
M3     ------------GAGCAGCACCGGGCCACG--GCGCAATCACCTAAAATCGGAGCCGCCGGGTG
M7     -GGAGGCCGAGAGGGCAGAACCCAGCGACGCGACGGAAAGACCATAACGGCAGC----------
M8     ------GCACACGGGCCGCACCC-----CT--GCGAAACGACTATACGTCACTAGAGCAGGTTT
M1A    GCACCTCGTTGAAGGAAG--------GACGGTGCGGAAAAAAAATAACGCACAACGCGGCA---
```

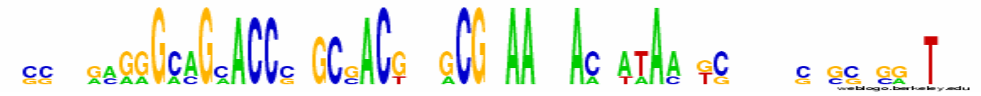

LOCAL SUPERMOTIF 2

```
M10     ----GTGCGAGCAGGGCAACCAGAAGAAAACGTAGGTAGAGTG
M7A     TTACGGATAGGCAAGGCAA---GGAGGAAACGATGCCGCGGGA
M13A    ---GTGACCAAGGTAGTAACCAAGAGAAAACGTGTTCCAGGGT
```

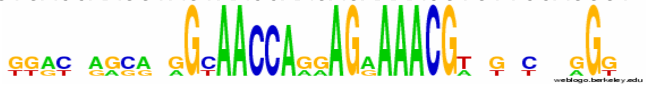

LOCAL SUPERMOTIF 3

```
M8      GCACACGGGCCGCACCCCTGCGAAACGACTATACG
M6A     -GCGCTGAGCCTCACGCCTGGGGACAAAACAGTTG
M11A    ----GTATGAGTCAGGCCTGGGAAAAGCAACGACG
```

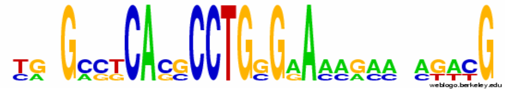

LOCAL SUPERMOTIF 4

```
M12     CAGACATCTACGTGGGCAGGGAGCATTG
M8A     CACTGAAATGTGTGGGCACATGGACAAG
M10A    TAGGGGGTGGCGTGGGCATTCTAAAGCG
```

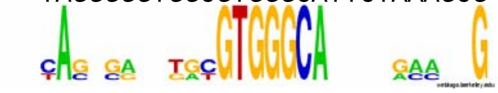

LOCAL SUPERMOTIF 5

```
M1      ---GATTAGGCGAACATAGTAGTGAAAGAAAGTACAGCATGAGGGGCT---------
M2      GTTGGTTACGCGTAC-----AGTGAAA--CTCGGAAAGTTGAAGGGCCAAATGGGAA
```

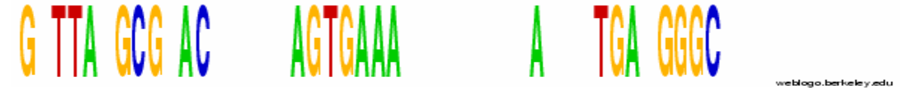

LOCAL SUPERMOTIF 6

```
M7      GAGGCCGAGAGGGCAGAACCCAGCGACG-CGACGGAAAGACCATAACGGCAGC
M8      ---GCACACGGGCCGCACCCCTGCGAAA-CGACTATACGTCACTAGAGCAGGT
M10A    TCGTAGGGGGTGGCGTGGGCATTCTAAAGCGGCTAGGCGTGACCCGGAG----
M11A    AAGCAACGACGGGCGTAGGCATACAAAA-CGTTCGTTGGCAATGT--------
```

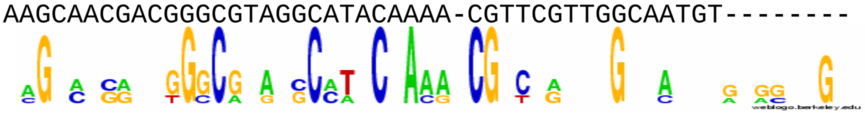

LOCAL SUPERMOTIF 7

```
M10     GGGCAACCAGAAGAAAACGTAGGTAGAGTG
M6A     CACGCCTGGGGACAAAACAGTTGCAAATGG
M11A    GGCGTAGGCATACAAAACGTTCGTTGGCAA
M13A    TAGTAACCAAGAGAAAACGTGTTCCAGGGT
```

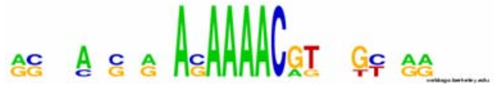

LOCAL SUPERMOTIF 8

```
M1     -----AGTGAAAGAAAGTACAG-CATGAGGGGCT------
M7     GGAGGCCGAGAGGGCAGAACCCAGCGACGCGACGGAAAGA
M10    ----GTGCGAGCAGGGCAACCA-GAAGAAAACGTAGGTAG
M11    AAGAGCGGAGAACGTTGAACCGACCGGAGG----------
M13    ------GCGAACGAGACAACCA-GATAAGGACCGCCCAAT
M1A    -----CGTTGAAGGAAGGACGGTGCGGAAAAAAAATAACG
M8A    ------ATGGACAAGCGACCCA-CATACGCTGTGGGCACA
```

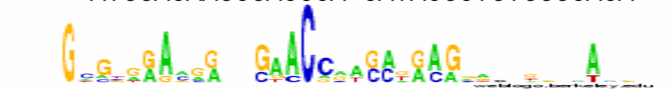

LOCAL SUPERMOTIF 9

```
M5     ------------GG-TACCGAAAAGTCATTGGTTACAACCAGGCAGGCGCAGGAA
M1A    TGAAGGAAGGACGG-TGCGGAAAAAAAATAACGCACAACGCGGCA----------
M6A    GCTGAGCCTCACGCCTGGGGACAAAA-CAGTTGCAAATGGCGTTGACAA------
```

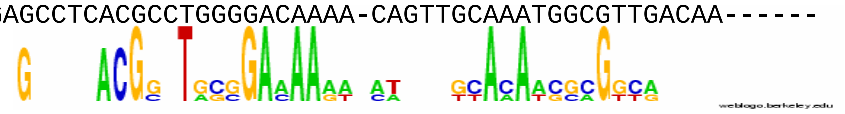

LOCAL SUPERMOTIF 10

```
M1     GTGAAAGAAAGTACAGCATGAGGGGCT---
M3     GAGCCGCCGGGTGCAGCATGTGAC------
M6     TGAGCGACTGAGGCAGCATGGAATCATACG
```

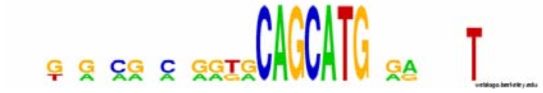

Supplementary Figure 1A

B

```
         LOCAL SUPERMOTIF 1
WL9     ACACGGAGGCACCTACAGTACTGGGGCAGGAGTTGCGCAGAGGACTAGTGGAAAAGGAGT
WL22    ACACGGAGGCACCTACAGTACTGGGGCAGGAGTTGCGCAGAGGACTAGTGGAAAAGGAGT
```

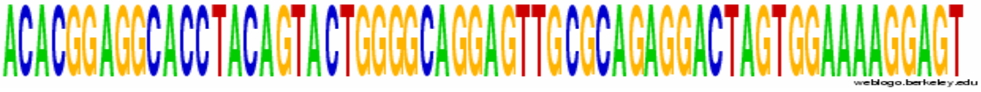

```
         LOCAL SUPERMOTIF 2
WL9     CACCTACAGTACTGGGGCAGGAGTTGCGCAGAGGAC--T------AGTGGAAAAGGAGT----
WL14    -----GAGACAAGTCGGTAGGAC-AGAGGAGTGAACAAAAGAAGCAGAGAGGAGAGTCAGAGC
WL16    AGAAATATAAACGTGGGGAGGACCAGGGGAGAGTAC--TCCAAAGAGTGAAGAAGGTT-----
WL22    CACCTACAGTACTGGGGCAGGAGTTGCGCAGAGGAC--T------AGTGGAAAAGGAGT----
```

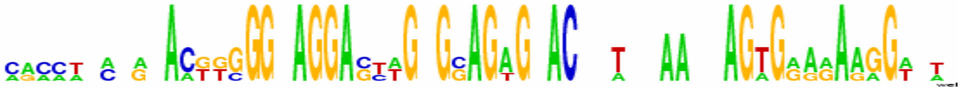

```
         LOCAL SUPERMOTIF 3
WL1        ATACACAGAACAGAGGAGAAGGGGATGAATCAATA
WL9        AGGAGTTGCGCAGAGGACTAGTGGAAAAGGAGT--
WL16       TATAAACGTGGGGAGGACCAGGGGAGAGTACTCCA
WL22       AGGAGTTGCGCAGAGGACTAGTGGAAAAGGAGT--
```

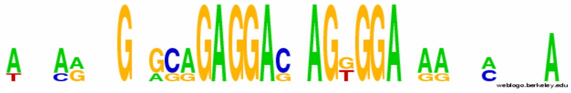

```
         LOCAL SUPERMOTIF 4
WL3        ACCATTGGTCAGTGGCAGAGGTTCATCACCGTC
WL9        GCAGAGGACTAGTGGAAAAGGAGT---------
WL11       GACGTAAAGAAGACGAAAGGGAGAACATTCGGG
WL13       GGTGTGCGTGAGTTGAAAAGGCAGGGCAA----
WL18       ---------------AAAAGGACAGGAGC----
WL22       GCAGAGGACTAGTGGAAAAGGAGT---------
```

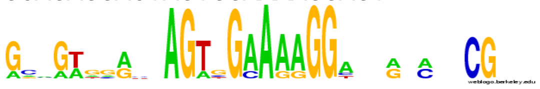

```
         LOCAL SUPERMOTIF 5
WL1     ATGAGAATA------CACAGAACAGAGGAGAA-G---GGGATGAATCAATAAGGTCACACTAACCAA
WL2     AAGTGTGTA------CACTAAAAAGCTAGCCCCC---GGAAAGAA-------CGTCAACAAAAGTAA
WL6     -------CGGAAGAGCACAAAAAAGGGGACTCGCCTTGGAAGGAA-------CCTGC----------
WL11    ----------GCCAGCACGACAAAGAGCAGGA-C---GTAAAGAAGACGAAAGGGAGAACATTCGGG
```

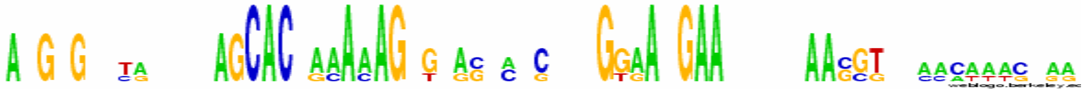

```
         LOCAL SUPERMOTIF 6
WL1  GAGAATACAC---AGAACAGAGGAG-----A--AGGGGATGAATCAATAAGGTCACACTAACcaacac-
WL7  aggccTAAAC---GAATGAACGGGG-----A--AAGTAAGGAATGATTGGGACCATACAcagt------
WL11 -------GCCAGCACGACAAAGAGCAGGACG-tAAAGAAGACGAAAGG-GAGaacattcggggagacga
WL14 GAGACAAGTCGGTAGGACAGAGGAGTGAACA--AAAGAAGCAGAGAGGAGAGTCAGAGCAGC-------
WL21 actATAAGTCAG---ATGGAGGGGG-----AgcATGAAACAATTGTTTAAGAACGTAAAatggctga--
```

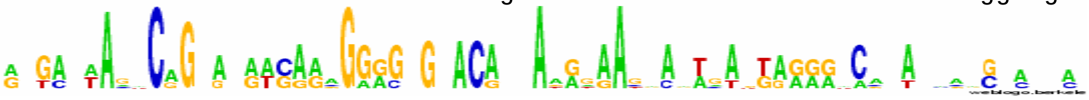

```
         LOCAL SUPERMOTIF 7
WL2     ------AAAAATAAAGTGTGTACACTAAAAAGCTAGCCCCCGGAAAGAACGTCAACAAAAGTAAGT
WL5     ACACCTGGAATTAAATTCAGGACACCCAA---CGCGCCTACGAATAGAGCGGGAACGACGACC---
```

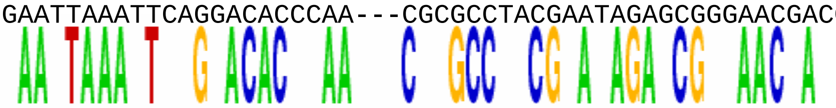

LOCAL SUPERMOTIF 8

```
WL1            CACAGAACAGAGGA-GAAGGGG---ATGAATCAATAAGGTCACAC
WL21           ACTATAAGTCAGATGGAGGGGGAGCATGAAACAATTGTTTAAGAA
```

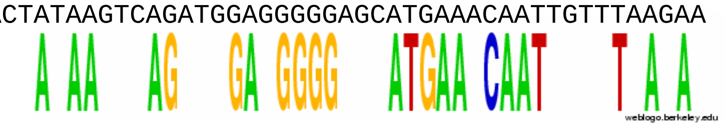

LOCAL SUPERMOTIF 9

```
WL1            --------ATGAGAATACACAGAACAGAGGAGAAGGGGATGAATCA
WL16           AGGACCAGGGGAGAGTACTCCAAAGAGTGAAGAAGGTT--------
```

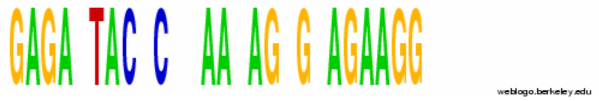

LOCAL SUPERMOTIF 10

```
WL2  ---AAAAATAA------------------------------AGTGTGTACACTAAAAAGCTAGCCCCCG
WL6  ----------------------------------------CGGAAGAGCACAAAAAAGGGGACTCGCCTT
WL14 GGTAGGACAGA------------------------------GGAGTGAACAAAAGAAGCAGAGAGGAGA
WL16 ----------CGCAAGAAATATAAACGTGGGGAGGACCAGGGGAGAGTACTCCAAAGAGTGAAGAAGGT
```

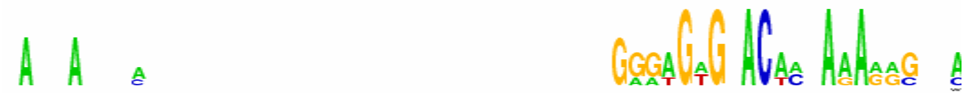

Supplementary Figure 1B

C

LOCAL SUPERMOTIF 1

```
LS12      -----GGTAAGGTGGAAGGGAAAAGGAAA-----CGGAATGAACCCGCGTTATGG
LS1B      ---------AAGTGAGAGGCAAAAGGAAAAAAAAAGGTGAACAAGGCTGCTC----
LS5       TCGGCGGAGGAGTGGGAAGAAAAACGAGAGAAGAGTTGGAACG-----------
LS5B      ------------GCAGGGGGAAAGGGA--TTGAACGAGAAGAAGGAGGAGC----
LS8       AGGGCAGCGGCGA-GAAGGGGAAA---AA-----AGAAAAGAACTATTCGTGGAG
```

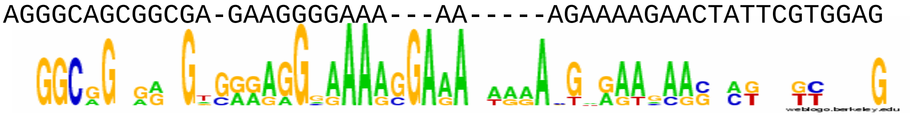

LOCAL SUPERMOTIF 2

```
LS1B   CCGCACACATAAGGG---------------------GGAGGGAAAGACAGCAGAATAAAGAGGAG
LS12   GGTAAGGTGGAAGGG---------------------AAAAGGAAACGGAATGAACCCGCGTTATG
LS1B   ----------AAGTG---------------------AGAGGCAAAAGGAAAAAAAAAGGTGAACAA
LS5    ----------GCAAACGGGGGCGTTTCGGCGGAGGAGTGGGAAGAAAAACGAGAGAAGAGTTGGA
LS5B   ----------------------------------GCAGGGGGAAAGGGATTGAACGAGAAGAAGG
LS8    ------------CCTGGAGGAGGGCAGCGGCGA-GAAGGGGAAAAAAGAAAAGAACTATTCGTGG
```

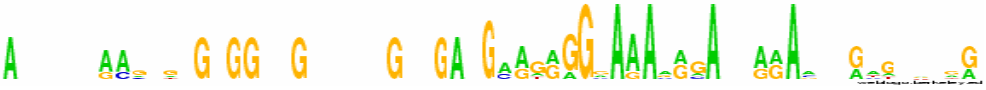

LOCAL SUPERMOTIF 3

```
LS1B   GCCGCACACATAAGGGGGAGGGAAA--GACAGCAGAATAAAGAGGAGGCGATAGTGC-----
LS12   --------GGTAAGGTGGAAGGGAAAAGGAAACGGAATGAACCCGCGTTATGGCCGAAAAGG
LS5B   ---------GCAGGGGGAAAGGGAT--TGAACGAGAAGAAGGAGGAGC--------------
LS9    ---GGGGCTTCATGAGAGAAGGAAC--CACAGACGAAAAAAAGGGGGGCGTTCAAGAGGAAG
```

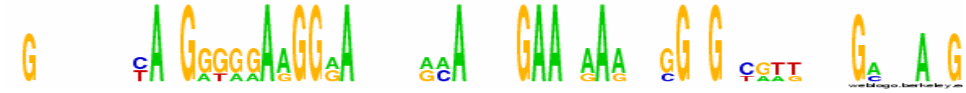

LOCAL SUPERMOTIF 4

```
LS10   -------ATTAACGAGATGAAGGAAACGGGAGGGAG
LS5B   AAAGGGATTGAACGAGAAGAAGGAGGAGC-------
LS9    AGGAACCACAGACGAAAAAAAGGGGGGCGTTCAAGA
LS9B   TAAGCTTGCGTCGAAGATGAAGAAAATGTCGAATCC
```

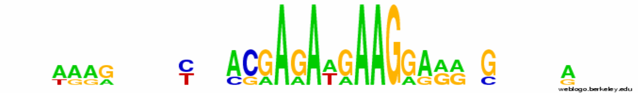

LOCAL SUPERMOTIF 5

```
LS10    ---GAGGGA--GACAGGCACAACGGCGACAGAAAAGAGAGAGTGC----
LS1B    TAAGGGGGAGGGAAAGACAGCAGAATAAAGAGGAGGCGATAGTGC----
LS15B   CGAGGACGA--AAGAGTTTAGAAAAGGAGACGGCGCTTCGAACGAACCA
LS5B    GCAGGGGGA--AAGGGATTGAACGAGAAGAAGGAGGAGC----------
LS7B    TGAGGGGAG--GTTGGCGCGAAGGCGAGGCAGCACGACCAGAAGTTTTT
LS8     ---GAGGGC--AGCGGCGAGAAGGGGAAAAAAGAAAAGAACTATTCGTG
```

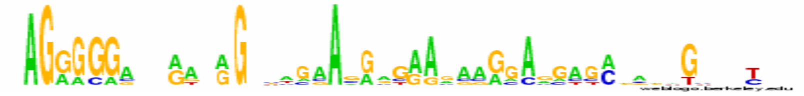

LOCAL SUPERMOTIF 6

```
LS12    AGGTGGAAGGGAAAAGGAAACGG-------AATGAACCCGCGTTATGG
LS15B   AAAGAGTTTAGAAAAGGAGACGGCGCTTCGAACGAACCATCCCCTAGG
```

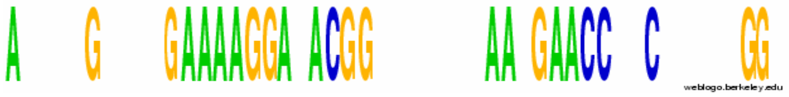

LOCAL SUPERMOTIF 7

```
LS10   CGAGATGAAGGAAACGGGAGGGAGACAGGCACAACGGCGA-----CAGAAAAGAGAGAGTGC------
LS5    ---------GCAAACG---GGGGCGTTTCGGCGGAGGAGTGGGAAGAAAAACGAGAGAAGAGTTGGAA
LS7B   ------------TGAG---GGGAGGTTGGCGCGAAGGCGAGGCAGCACGACCAGAAGTTTTTGAGTTC
LS9    ACAGACGAAAAAAAGG---GGGGCGTTCAAGAGGAAGAGACCGA-----------------------
```

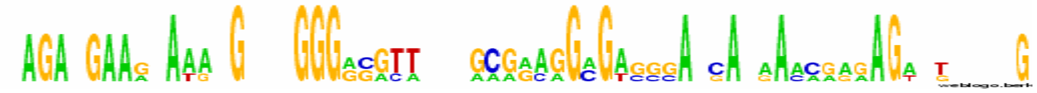

LOCAL SUPERMOTIF 8

```
LS10           -ATTAACGAGATGAAGGAAACGGGAGGGAGACAG
LS1B           ACAGCAGAATAAAGAGGAGGCGATAGTGC-----
LS12           AGGTGGAAGGGAAAAGGAAACGGAATGAACCCGC
LS15B          AAAGAGTTTAGAAAAGGAGACGGCGCTTCGAACG
LS8B           ----------GAAAAGGAGGGGATTTGAAATCGC
LS9            GGGGGGCGTTCAAGAGGAAGAGACCGA-------
```

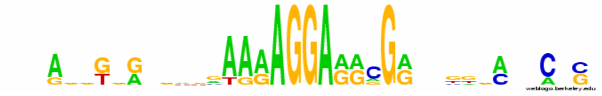

LOCAL SUPERMOTIF 9

```
LS1B           CACACATAAGGGGGAGGGAAAGACAG--C--AGAATAAAGAGGAGGCGAT
LS1B           AAGTGAGAGGCAAAAGGAAAAAAAAG--G--TGAACAAGGCTGCTC----
LS8            GGGCAGCGGCGAGAAGGGGAAAAAAGAAA--AGAACTATTCGTGGAGCGG
LS9            GAAGGAACCACAGAC-G-AAAAAAAG--GGGGGCGTTCAAGAGGAAGAGA
```

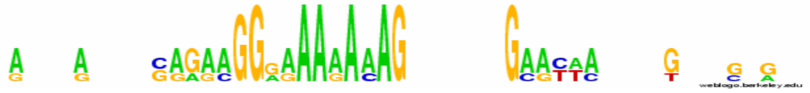

LOCAL SUPERMOTIF 10

```
LS12    -----ggtaaggtggaaggGAAAAGGAAACGGAATGAACCCGCGTTATGGCCGAAaagggac-----
LS4b    -ggccCGACGA------------AGTGGGGGGAGGAACGTCGTGTTGAGTGATTGGAgtccctgggc
LS5     cgtttCGGCGG------------AGGAGTGGGAAGAAAAACGAGAGAAGAGTTGGAAcg--------
LS8b    -------------------GAAAAGGAGGGGATTTGAAATCGCGTGAAAGGTGAAttgtttgccagg
```

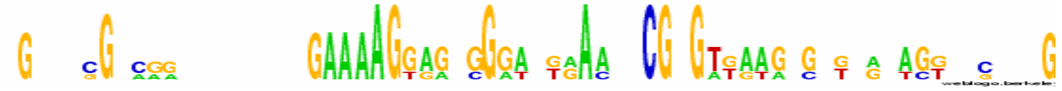

Supplementary Figure 1C

Supplementary Figure 2.

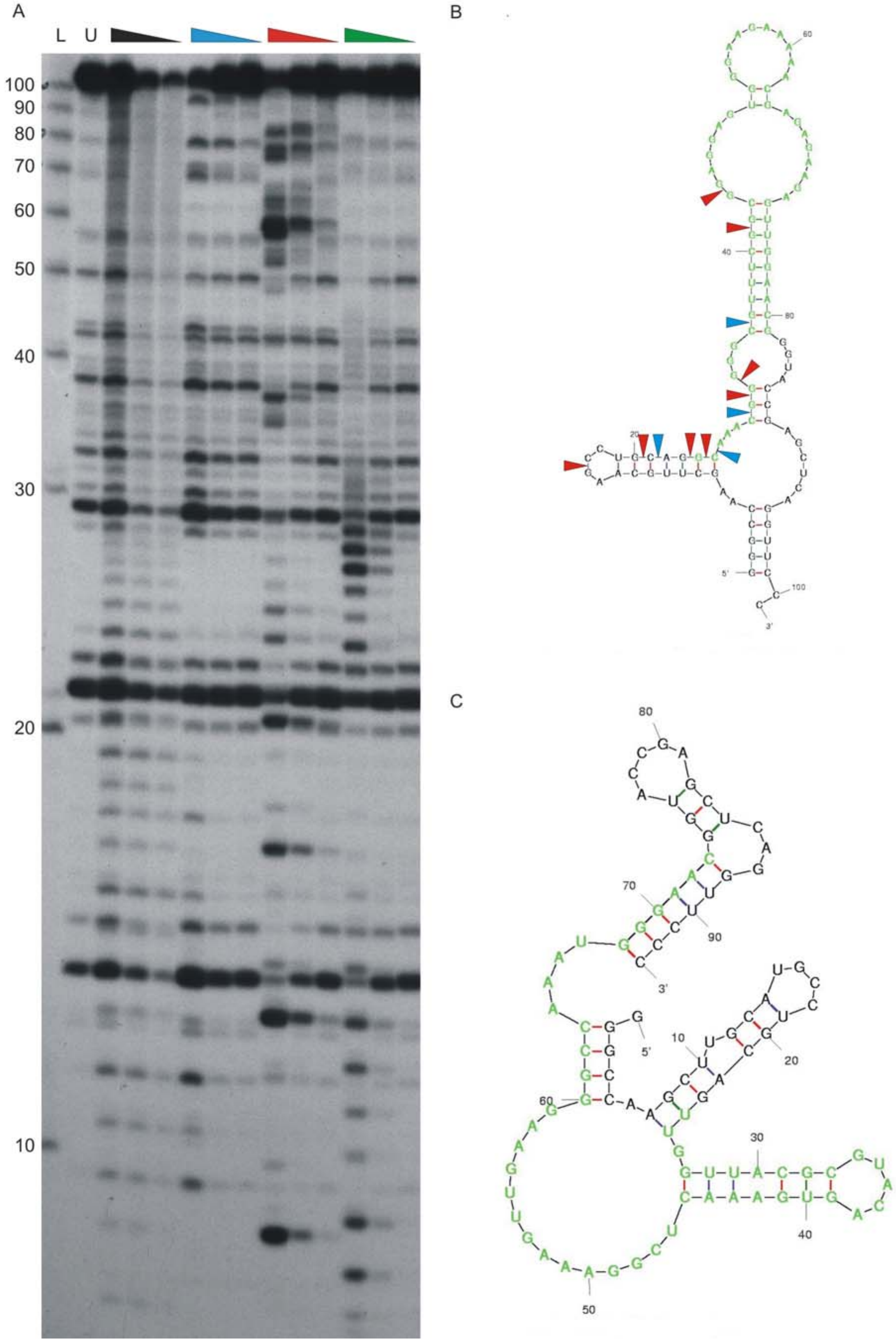

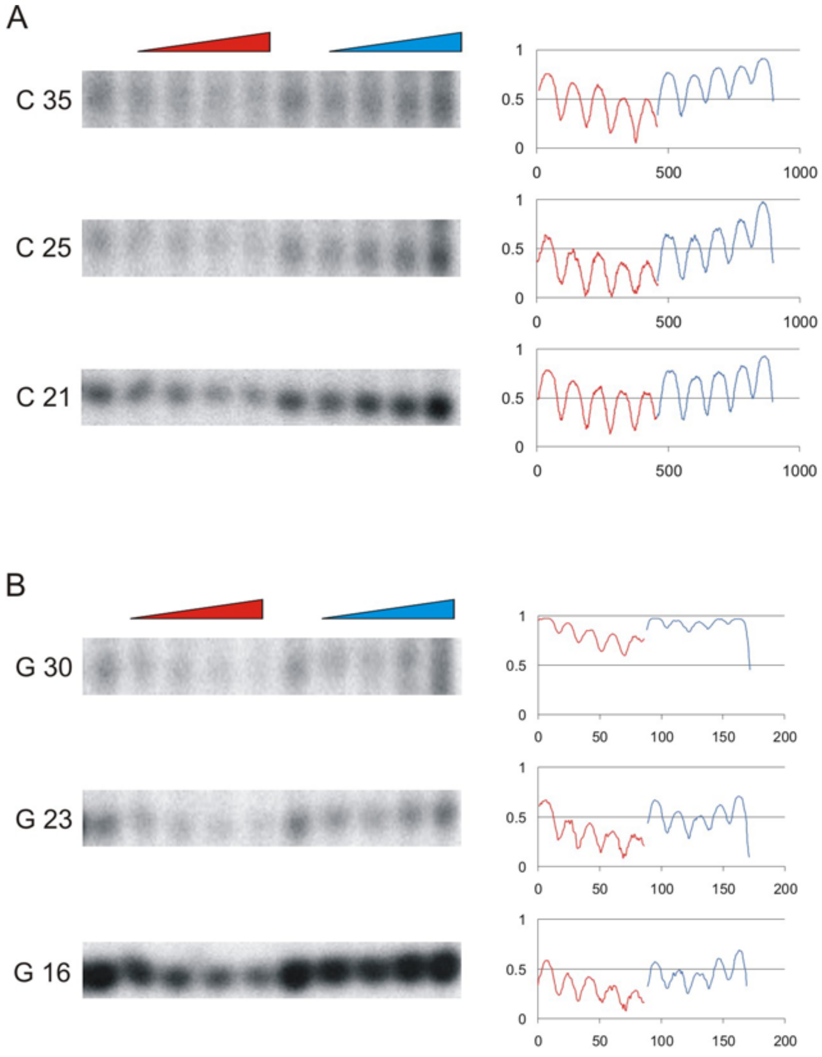

Supplementary Figure 3.

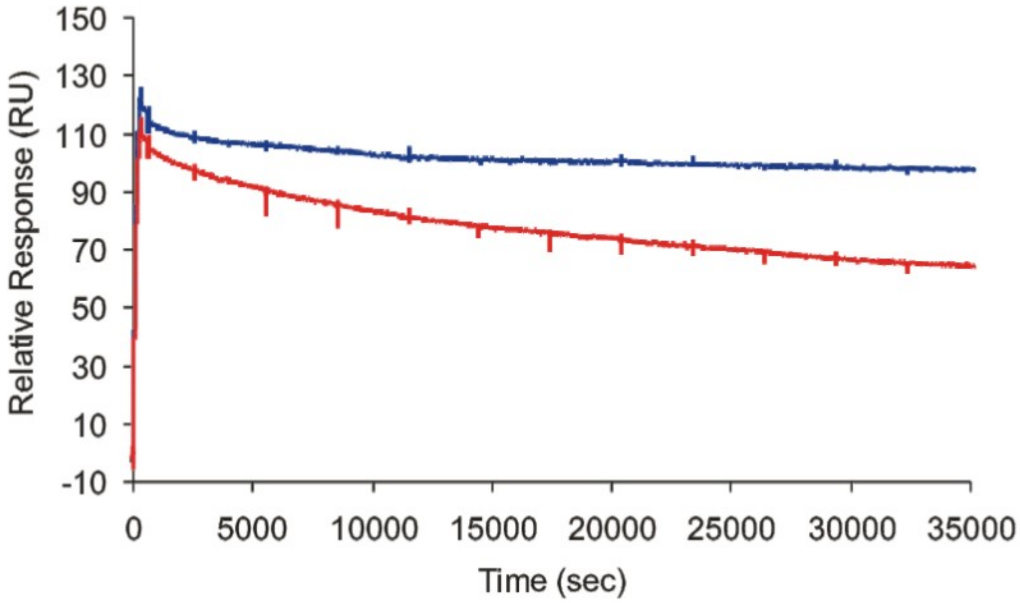

Supplementary Figure 4.

Supplementary Table 1. Affinities of the aptamers derived from the SPR assay.

Aptamer	Target	Apparent dissociation rate constant		Apparent association rate constant		Apparent equilibrium dissociation constant					
**WL-2**		k_{diss} *(1/sec)*	*SE(kd)*	k_{ass} *(1/M.sec)*	*SE(ka)*	K_D (M)	*Rmax (RU)*	*SE(Rmax)*	*RI (RU)*	*SE(RI)*	*χ2*
	Monomer	0.0504	5.50E-02	9.45E+03	3.51E+04	5.34E-06	16.4	36.2	17.1	0.952	
	WL	8.37E-04	1.13E-05	1.12E+05	1.26E+03	7.48E-09	593	2.8	426	1.29	4.73E+00
	LS	8.42E-04	9.10E-06	1.10E+05	983	7.63E-09	754	2.62	503	1.29	
**M-2**		k_{diss} *(1/sec)*	*SE(kd)*	k_{ass} *(1/M.sec)*	*SE(ka)*	K_D (M)	*Rmax (RU)*	*SE(Rmax)*	*RI (RU)*	*SE(RI)*	*χ2*
	Monomer	8.13E-03	1.06E-03	7.10E+05	9.75E+04	1.15E-08	34.4	1.89	47.6	0.905	
	WL	7.48E-04	3.12E-05	1.76E+05	8.04E+03	4.25E-09	766	26.7	155	0.855	9.10E-01
	LS	3.55E-03	5.59E-05	5.21E+05	1.24E+04	6.82E-09	279	3.12	207	0.89	
**LS-5**		k_{diss} *(1/sec)*	*SE(kd)*	k_{ass} *(1/M.sec)*	*SE(ka)*	K_D (M)	*Rmax (RU)*	*SE(Rmax)*	*RI (RU)*	*SE(RI)*	*χ2*
	Monomer	8.16E-03	1.96E-04	4.78E+04	3.53E+03	1.71E-07	30.1	0.746	15.2	0.578	
	WL	6.71E-04	1.48E-05	2.45E+04	227	2.74E-08	285	1.04	63.4	0.458	2.39E+00
	LS	3.97E-04	1.15E-05	1.79E+04	169	2.21E-08	411	1.91	57.5	0.436	
